# Moving beyond the scale: musculoskeletal risks, evidence gaps and emerging combination strategies to optimize the quality of weight loss pharmacotherapy in older adults

**DOI:** 10.3389/fragi.2025.1640030

**Published:** 2025-10-24

**Authors:** Kieran F. Reid, Shalender Bhasin

**Affiliations:** ^1^ Laboratory of Exercise Physiology and Physical Performance, Brigham and Women’s Hospital, Harvard Medical School, Boston, MA, United States; ^2^ Research Program in Men’s Health: Aging and Metabolism, Boston Claude D. Pepper Older Americans Independence Center for Function Promoting Therapies, Brigham and Women’s Hospital, Harvard Medical School, Boston, MA, United States

**Keywords:** obesity pharmacotherapy, older adults, musculoskeletal health, lean mass preservation, promyogenic therapy, exercise interventions, multimodality interventions

## Abstract

The increasing prevalence of obesity among older adults is a significant clinical and public health challenge. In this population, obesity contributes to numerous chronic diseases, functional decline and elevated mortality. This growing concern highlights the urgent need for more effective weight management strategies for older adults. Pharmacologic treatments, particularly GLP-1 receptor agonists and dual agonists, have emerged as promising treatments for weight loss, but their effects remain understudied in older adults. In this article, we discuss the potential musculoskeletal implications associated with the use of weight loss pharmacotherapy among older adults. We emphasize the consequences of lean mass loss, particularly the loss of skeletal muscle mass, which represents a critical determinant of ambulation, physical function and major regulator of metabolic health in older adults. We also describe the adverse risks of weight regain and weight cycling, and the significance of lean mass preservation during weight loss for older individuals. Finally, we identify knowledge gaps associated with safe and effective obesity pharmacotherapy in older adults and emphasize the potential benefits of combining GLP-1 therapies with promyogenic agents, structured exercise, and targeted nutritional interventions for optimizing weight loss quality in this population. These integrated approaches merit further investigation in clinical trials to determine their synergist effects for enhancing body composition while promoting independence, vitality and wellbeing in older adults undergoing pharmacologic weight loss.

## Introduction

The rising prevalence of obesity among older adults, now affecting over 40% of individuals aged 65 and older in the United States, represents a significant clinical and public health challenge (Fakhouri et al., 2012; Hales et al., 2020). In this population, obesity contributes to numerous chronic conditions, including type 2 diabetes, cardiovascular disease, many types of cancers, osteoarthritis, and neurodegenerative disorders, and is independently associated with functional decline, increased disability, greater healthcare utilization, and elevated mortality (Villareal et al., 2005; Guh et al., 2009). This escalating burden highlights the urgent need for more effective weight management strategies for older adults.

Pharmacologic therapies, particularly glucagon-like peptide-1 receptor agonists and newer dual agonists (GLP-1s), have emerged as promising treatments for obesity. These agents induce rapid and substantial weight loss, with accompanying improvements in glycemic control and cardiometabolic outcomes (Kushner et al., 2020; Rubino et al., 2021). However, a key limitation of the current evidence base is the underrepresentation of older adults in clinical trials of pharmacologic weight loss therapies. As a result, there is limited understanding of how these medications specifically impact older adults, particularly in relation to their long-term effects on musculoskeletal health, physical functioning and other outcomes vital to maintaining quality of life, independence, and healthspan among older persons.

This article highlights current knowledge gaps associated with the growing use of weight loss medications in older adults and potential strategies for quality weight management. We discuss the consequences of lean mass loss, the adverse implications of weight regain and weight cycling, and the significance of lean mass preservation during weight loss in older individuals. We also identify critical areas for future research and highlight emerging adjunctive or combination strategies that may minimize adverse risks and optimize the effects of GLP-1-induced weight loss approaches among older adults (Table 1).

**TABLE 1:** Key evidence gaps and future research directions to improve the quality of pharmacologic weight loss in older adults.

Evidence gaps	Opportunities for future research
Older adults, especially those with functional limitations, remain underrepresented in trials of GLP-1 receptor agonists and other weight loss pharmacotherapies	Design and implement randomized trials that intentionally recruit diverse older populations, including those with multimorbidity or reduced physical function
Insufficient data on the impact of pharmacologic weight loss on body composition, physical function, and bone health in functionally limited older adults	Incorporate detailed assessments of lean and bone mass, muscle performance, physical function and fall risk in future pharmacotherapy studies
Long-term consequences of weight regain and weight cycling following GLP-1 treatment are poorly characterized in older populations	Conduct longer term studies to examine the musculoskeletal and metabolic implications of repeated weight loss and regain
Uncertainty about the additive or synergistic effects of combination strategies to mitigate musculoskeletal risk during pharmacologic weight loss	Evaluate multimodal interventions that combine GLP-1 therapy with exercise training, anabolic therapies, and/or targeted nutrition interventions

## Musculoskeletal risks associated with lifestyle weight loss strategies in older adults

Lifestyle-based interventions, including caloric restriction, exercise training, and behavioral support, have demonstrated meaningful short-term benefits for older adults with obesity. These include reductions in fat mass, improved glycemic control, enhanced mobility, and better quality of life (Rejeski et al., 2011; Villareal et al., 2011; Waters et al., 2013; Nicklas et al., 2019). However, nearly all weight loss strategies, whether intentional or unintentional, are accompanied by loss of lean mass, and particularly the loss of skeletal muscle mass. Skeletal muscle mass is essential not only for physical function and everyday activities (Reid and Fielding, 2012) but also as a pivotal regulator of systemic metabolic health. It serves as a primary site for glucose uptake and utilization, influences adipose tissue regulation, and is a major determinant of resting and total energy expenditure (Basaria and Bhasin, 2012). Consequently, the implications of skeletal muscle loss are particularly important for older adults, who may already be nearing a threshold of vulnerability for musculoskeletal complications such as sarcopenia, frailty, and mobility impairment. Furthermore, reduced appendicular muscle mass and muscle strength are powerful predictors of survival, physical functioning, and independence in older adults (Newman et al., 2006; Ferrucci et al., 2012; Cawthon et al., 2021; Wang et al., 2023). While low baseline muscle mass is a well-established risk factor for adverse outcomes, emerging evidence suggests that muscle loss occurring during periods of weight change may confer additional, independent risks. For example, findings from the Health ABC study indicate that weight loss in older adults significantly decreases thigh muscle area, independently predicting greater mortality risk (Santanasto et al., 2017). Therefore, the preservation of skeletal muscle mass should be a central objective in any weight reduction strategy, whether lifestyle-based or pharmacologic.

Other epidemiologic evidence highlights bone loss as another significant musculoskeletal consequence of weight loss in older adults. In the Study of Osteoporotic Fractures, women who lost ≥5% of body weight had a 68% higher risk of hip fracture and a 39% higher risk of non-spine fracture, independent of baseline body composition (Ensrud et al., 2003). These results were further supported by findings of the Women’s Health Initiative Study, reporting a 65% increased risk of hip fractures with unintentional weight loss ≥5% over an 11-year follow-up in a large cohort of postmenopausal women (Crandall et al., 2015). A sub-analysis of the Look AHEAD study found that participants in the lifestyle intervention group lost significantly more bone mineral density (BMD) at the hip, femoral neck, and lumbar spine than controls over 4 years, despite only modest (∼6.5%) weight loss (Lipkin et al., 2014). These results were further supported by Johnson et al., who reported an increased risk of frailty fracture with long term intentional weight loss over a median 9.6 years follow up period (Johnson et al., 2017).

## Adverse effects of weight regain and weight cycling

Another critical yet frequently overlooked aspect of obesity treatment in older adults is the pattern and composition of weight regain. Even when weight loss is intentional and initially beneficial, the long-term physiological consequences can vary substantially. Data from the Health ABC study demonstrate that lean mass regained after weight loss is consistently less than the amount originally lost, resulting in an asymmetrical pattern of recovery that favors fat accumulation (Newman et al., 2005). This imbalance may accelerate the development of sarcopenic obesity, a condition characterized by concurrent reductions in muscle mass and increases in adiposity which confers a greater risk for disability, hospitalization, and mortality than either obesity or sarcopenia alone (Zamboni et al., 2008; Batsis et al., 2014). This pattern of disproportionate fat regain can be further complicated by weight cycling which is defined as repeated episodes of intentional weight loss followed by unintentional weight regain. Lee et al. demonstrated that older adults undergoing repeated weight cycling experienced a net loss of lean mass over time, reinforcing concerns about the cumulative musculoskeletal toll of weight fluctuation in aging populations (Lee et al., 2010). Middle-aged and older adults who experience weight cycling or weight regain after intentional weight loss exhibited greater deficits in muscle performance and other objective measures of physical function compared to those with more stable weight trajectories over an ∼8-year follow-up period (Beavers et al., 2015). Furthermore, weight cycling is associated with greater adipose tissue inflammation and insulin resistance (Anderson et al., 2013; Johansson et al., 2014), and higher all-cause mortality than a consistently obese state (Oh et al., 2019).

## Critical evidence gaps associated with pharmacologic weight loss in older adults

While GLP-1s are emerging as transformative agents in obesity treatment, their impact on body composition, particularly in older adults, remains insufficiently understood. Many pivotal trials primarily enrolled middle-aged adults and either underrepresented older adults or excluded those over the age of 65 altogether, restricting generalizability to the aging population. In addition, most trials to date have prioritized endpoints that focus on the magnitude of total weight loss and cardiometabolic outcomes, with limited focus on the composition of actual weight lost or on functional outcomes of meaningful relevance to older adults. Pooled data suggest that lean mass loss may account for approximately 40%–60% of total weight lost with GLP-1 therapy (Neeland et al., 2024). Subgroup analyses from STEP 1 and SURMOUNT-1 indicate that in participants aged ≥65, lean mass accounted for approximately 40% and 26% of total weight lost, respectively (Wilding et al., 2021; Jastreboff et al., 2022; Look et al., 2025). While informative, these data are based on small samples and do not adequately capture the functional implications of weight loss in broader, more heterogeneous populations of older adults, and particularly among those who may have multiple comorbidities, or who are at elevated risk for sarcopenia, frailty falls, or other aging-related conditions.

## Combination strategies to optimize the quality of pharmacologic weight loss

Given the adverse musculoskeletal risks associated with both lifestyle- and pharmacologically induced weight loss in older adults, growing attention is being directed toward combination strategies aimed at optimizing body composition changes while preserving physical function. Several investigational promyogenic agents (pharmacologic compounds designed to stimulate muscle growth or inhibit muscle catabolism) are emerging as promising in this domain. Bimagrumab, a monoclonal antibody that inhibits activin type II receptors, has been shown to simultaneously decrease fat mass and increase lean mass. In a randomized controlled trial involving older adults with obesity, 48 weeks of bimagrumab treatment led to a 20% reduction in fat mass and a 3.6% increase in lean mass, alongside favorable reduction in hemoglobin A1c (Heymsfield et al., 2021). Other agents such as trevogrumab and garetosmab, targeting myostatin and activin pathways, have demonstrated similar anabolic potential (Stefanakis et al., 2024). Selective androgen receptor modulators, including enobosarm, have also shown promise for preserving lean mass during weight loss. Early-phase trials of enobosarm reported improvements in strength and muscle mass among older adults undergoing cancer-induced muscle wasting (Fonseca et al., 2020). More recently, the Phase 2 b QUALITY trial reported that combining enobosarm with semaglutide reduced lean mass loss by 71% and yielded a 27% greater reduction in fat mass compared to semaglutide alone. Notably, this combination also led to superior preservation of stair-climbing power and lower-extremity strength, highlighting its functional benefits.

Exercise remains one of the most effective adjunctive strategies to preserve musculoskeletal health in weight loss clinical trials. Nicklas et al. evaluated the effects of resistance training with and without caloric restriction in older adults (Nicklas et al., 2015). They found that participants who combined resistance training with caloric restriction experienced superior improvements in mobility, knee strength, and self-reported disability, despite modest losses in lean mass (18%–20% of the total weight lost). These findings were reinforced by a randomized controlled trial by Villareal et al., which compared the effects of aerobic exercise, resistance training, and a combination of both among obese older adults undergoing weight loss (Villareal et al., 2017). The study found that while all groups experienced improvements in functional status, the combination of aerobic and resistance training led to the greatest improvements in physical performance, preservation of lean mass, and attenuation of bone loss.

Building on this foundation of evidence from lifestyle-based interventions, recent pharmacologic weight loss trials have evaluated the combination of GLP-1 therapy with exercise training. Lundgren et al. demonstrated that combining exercise with liraglutide resulted in superior maintenance of weight loss, improved physical performance, and greater preservation of muscle mass compared to either intervention alone (Lundgren et al., 2021). Further supporting this combined approach, a secondary analysis of a randomized clinical trial involving adults with obesity found that the combination of exercise and GLP-1 therapy preserved BMD at the hip, spine, and forearm, despite greater total weight loss (Jensen et al., 2024). In contrast, GLP-1 treatment alone was associated with significant reductions in hip and spine BMD compared to either exercise alone or placebo. In a recent review article, Locatelli et al. further endorse the approach of combining GLP-1 therapy with exercise training, specifically emphasizing that resistance training may have significant potential to optimize body composition, preserve lean mass, and mitigate the muscle loss typically observed with pharmacologic therapies (Locatelli et al., 2024). While the benefits of exercise are well established under controlled conditions, translating these effects into real-world settings can be challenging due to variability in long-term, program accessibility, and sustained engagement, particularly among older adults.

Adjunct nutritional interventions may offer additional support for preserving musculoskeletal health during pharmacologic weight loss in older adults. Campbell et al. emphasize the critical role of dietary protein in maintaining skeletal muscle, highlighting increased protein requirements in older populations due to age-related anabolic resistance (Campbell et al., 2023). Beta-hydroxy beta-methylbutyrate (HMB) supplementation has also shown potential for preserving appendicular lean mass and improving physical function during periods of weight loss, particularly when combined with resistance exercise (Li et al., 2025). Similarly, leucine-enriched protein supplements have shown beneficial effects on muscle protein synthesis in older adults (Katsanos et al., 2006).

Collectively, these emerging strategies offer an encouraging framework for optimizing the *quality* of weight loss in older adults - shifting the focus from monotherapies toward integrated, multimodal interventions that combine GLP-1 treatment with promyogenic drugs, structured exercise or targeted nutrition interventions. Combinations of these approaches warrant further investigation in clinical trials to determine whether they produce synergistic benefits for enhancing body composition, preserving lean mass and physical function, and ultimately promoting independence and healthspan in older adults undergoing pharmacologic weight loss.

## Summary and future directions

Even modest, intentional weight loss in older adults can lead to unintended musculoskeletal consequences, underscoring the importance of preserving skeletal muscle mass, as a fundamental component of effective obesity treatment in aging populations. Skeletal muscle determines physical functioning and is a major regulator of metabolic health, which provides a strong rationale for combining promyogenic agents with GLP-1-based pharmacologic therapies. However, despite the growing use of GLP-1 pharmacotherapy, there remains a lack of generalizable data on their short or long-term impact on fat-to-lean mass composition, physical function, fall and fracture risk, and other health outcomes in older adults. There is also a distinct need to conduct clinical trials that target older adults at elevated risk for musculoskeletal decline and generate evidence that can inform safe and effective obesity pharmacotherapy in this population. Weight cycling characterized by weight loss followed by weight gain is associated with adverse health outcomes. In this respect, future trials should incorporate more precise body composition measures and physical function endpoints such as strength, power, walking speed, fall risk, and mobility disability. Given the high prevalence of polypharmacy in older populations, future research should also consider the potential risks and cumulative burden associated with combining multiple pharmacologic agents, carefully balancing benefits in body composition and function with overall medication safety and tolerability. Future clinical trials are also needed to better understand the longer-term musculoskeletal consequences of GLP-1 therapy in older adults. The emphasis of GLP-1 therapy among older adults must shift from a singular focus on the magnitude of weight loss to the *quality* of weight loss–more pertinently defined as the preservation of muscle, bone, physical performance and other outcomes of meaningful importance to the wellbeing of older persons. The combination strategies that integrate GLP-1 pharmacotherapy with promyogenic drugs, exercise training, and nutritional strategies, represent promising avenues for research and clinical translation to ensure that weight loss improves, not compromises, independence, vitality, and wellbeing of older adults.

## Data Availability

The original contributions presented in the study are included in the article/supplementary material, further inquiries can be directed to the corresponding author.
